# Preparation of blueberry anthocyanin liposomes and changes of vesicle properties, physicochemical properties, in vitro release, and antioxidant activity before and after chitosan modification

**DOI:** 10.1002/fsn3.2649

**Published:** 2021-12-02

**Authors:** Lei Wang, Lulu Wang, Xi Wang, Baojun Lu, Jing Zhang

**Affiliations:** ^1^ College of Traditional Chinese Medicine Jilin Agricultural University Changchun China; ^2^ College of Medical Changchun University of Science and Technology Changchun China; ^3^ Hangzhou Mushannong Industrial Investment Co., Ltd Hangzhou China

**Keywords:** antioxidant activity, blueberry anthocyanin, chitosan, environmental stability, liposome, response surface methodology

## Abstract

The preparation of blueberry anthocyanin liposomes (BAL) was optimized by response surface methodology. Then, chitosan was used to modify BAL and the environmental stability, in vitro release, and antioxidant activity studies of anthocyanin liposome (An‐Lip), and chitosan‐modified anthocyanin liposome (CS‐An‐Lip) was studied. The results showed that the particle size, zeta potential, and entrapment efficiency of BAL were 210.7 ± 1.8 nm, ‐ 20.0 ± 1.0 mV, and 82.13%, respectively. After chitosan modification, the encapsulation efficiency and zeta potential of anthocyanin liposomes were improved. The results of environmental stability analysis showed that under certain conditions, the addition of chitosan could stabilize the color characteristics of anthocyanins and the loading amount of anthocyanins (LC%). In vitro release and simulated gastrointestinal digestion experiments showed that the addition of chitosan not only prolonged the sustained‐release time of anthocyanins, but also prolonged the residence time of anthocyanins in vivo, giving full play to the drug effect. In addition, the antioxidant activity test results showed that CS‐An‐Lip increased the antioxidant activity of anthocyanins.

## Graphical abstract

Blueberry anthocyanins with high entrapment efficiency were successfully prepared in this paper. Blueberry anthocyanin liposomes were modified with biopolymer chitosan, and the stability was improved after modification. It provides a basis for further research and identification of blueberry anthocyanins.

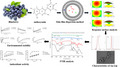

## 1 INTRODUCTION

Blueberry is well known as the “king of berries” and recommended by FAO (Food and Agriculture Organization) as one of the top five healthy fruits in the world (Joshi et al., 2016). It is rich in phytochemicals such as flavonoids, polyphenols, organic acids, and anthocyanins. Anthocyanins are considered to be the main component supporting the natural function of blueberries. Blueberry anthocyanin (BA) is a flavonoid polyphenol compound, which is composed of cyanidin and sugar molecules bound by glycosidic bonds (Casta et al., 2009). As a water‐soluble pigment, it has a wide range of pharmacological activities, including antioxidant, anti‐inflammatory, anti‐cancer, anti‐obesity, anti‐diabetic, and vision protection activities (Amini et al., 2017; Kamenickova et al., 2013). However, the biological application of anthocyanins is limited due to their extreme susceptibility to temperature, light, pH, oxygen, ascorbic acid, enzymes, and metal ions (Vemana et al., 2020). In recent years, how to improve the stability and bioavailability of anthocyanins has become a new research hotspot. Encapsulation is an important way to protect environmentally sensitive bioactive molecules (Shishir et al., 2018). So far, many carrier systems have been developed and used to encapsulate anthocyanins to enhance storage and gastrointestinal stability and to improve the bioavailability and delivery of anthocyanins at target points, such as micelles, dendrimers, complex condensates, cyclodextrins, solid lipid nanoparticles, cellulose nanocrystal, polyelectrolyte complex, nano liposome, and different polymer formulations (Cruz et al., 2019; Shaddel et al., 2018; Flores et al., 2015; Lin et al., 2019). In this study, we focused on the application of lipid‐based anthocyanin release system.

Liposomes are lipid‐based colloidal delivery system, which consist of one or more phospholipid bilayer membranes surrounding an aqueous core. Liposomes can be employed for the delivery of both hydrophobic and hydrophilic compounds even for dual drug delivery (Gomez and Fernandze, 2006). Liposomes are considered a promising and safe carrier because of their biocompatibility, amphiphilic, nontoxic, and non‐immunogenicity. Lipid molecules are easily biodegradable. It can enhance the transcellular transport by transient disruption of cellular lipophilic bilayers and could improve the para‐cellular drug transport through altering the tight junctions (Dumont et al., 2018). In recent years, liposome has a wide range of potential applications in drug delivery, cosmetics, and food formulations. But during storage, liposomes easily aggregate. The fusion of liposomal membranes can cause burst release of payload due to oxidation during storage and hydrolysis of phospholipid under low pH and enzymatic conditions (Shishir, Karim, Gowd, Xie, et al., 2019; Shishir, Karim, Gowd, Zheng, et al., 2019). So, how to effectively improve the function of liposome in complex environments became the focus of research. Some research suggests presence of glycolipids, glycoproteins, and proteins in the cell membrane helps to enhance its stability and function in the fluid‐mosaic membrane model (Nicolson, 2014). These coating materials can be attached to the surface of the liposome by covalent bonds, hydrogen bonds, van der Waals forces, hydrophobic interactions, and/or electrostatic interactions. Alternatively, the surfactant can be incorporated into the lipid bilayer itself to modify its surface properties, such as phospholipids, glycolipids, sterols, and surfactants (Seong et al., 2018).

Chitosan is a nontoxic, biodegradable polysaccharide. Modification of chitosan not only can slow the degradation of liposomes, but also prevents interplay among components during the process of storage and transportation. Due to the protonated amine groups on the repeated glycoside residues, chitosan has a cationic charge in an acidic medium and an increase in water solubility. The cationic nature of chitosan has attracted much attention as a polyelectrolyte excipient for medical use. In particular, the potential of chitosan as a carrier for anionic macromolecules (such as siRNA) has been highlighted by its effective intracellular and local delivery (Alavi et al., 2016; Zhang et al., 2018). In addition to electrostatic interactions with counterion molecules, chitosan has been reported to open tight junctions, alter the secondary structure of the stratum corneum keratin, and enhance cell membrane fluidity, thereby increasing skin permeability.

In this study, anthocyanin liposomes were prepared by thin‐film ultrasonic dispersion method, optimized by response surface method, established the preparation process, and verified the preparation process of anthocyanin liposomes by quadratic polynomial regression model. An‐Lip was characterized by microscope and particle size analyzer. The formation of An‐Lip was verified by infrared spectroscopy and then modified with chitosan. An‐Lip was used as control. The physicochemical properties of CS‐An‐Lip were determined by mean particle size, polydispersity index (PDI), zeta potential, and morphology. The environmental stability of liposomes under the conditions of temperature and time was investigated. The in vitro release, bioavailability, and antioxidant activity of the liposomes were further investigated. It provides a basis for the rational design of new liposome drug delivery system.

## 2 MATERIALS AND METHODS

### 2.1 Materials and chemicals

Blueberry anthocyanin was prepared in the laboratory, and its purity was 98% by HPLC. Soybean lecithin (SP) was purchased from Shanghai Everton Pharmaceutical Limited Company. Cholesterol (No.160612) was purchased from Beijing Biotechnology Limited Company. Chitosan (*Mw*.1.5 × 10^5^ Da. DDA ≥ 95%, viscosity 100–200 Mpa.s) was purchased from Xi'an Jingbo Biotechnology Limited Company. Phosphate‐buffered saline (PBS) was purchased from Sigma‐Aldrich Chemical Company. Double‐distilled water was used in all experiments, and all other chemicals were of analytical grade.

### 2.2 Preparation of An‐Lip and CS‐An‐Lip

All the liposomes were prepared by film ultrasonic dispersion method with some minor modifications (Sylvester et al., 2017). Firstly, SP and cholesterol were dissolved in chloroform (4 mL) and ether (8 mL) at the suitable ratio, and BA was dissolved in a phosphate buffer solution (PBS, 4 mL, pH7.3). The organic phrase was mixed completely by ultrasound in ice water to form stable water in oil type (W/O) emulsion. Secondly, the emulsion was placed in a round‐bottom flask and then evaporated to form a gel by using a rotary evaporator, and residual organic solvent was removed by pumping in a vacuum for 1–2 h. After that, BA solution was injected into round‐bottom flask, and then the resulting mixture was mixed uniform by using a rotary evaporator for 1 h; the mixture was homogenized using an ultrasonic cell disintegrator (JY92‐II DN, Xinzhi Bio‐technology and Science Inc.) for 20 min. Ultimately, PBS was injected into the mixture and rotary evaporation for 30 min, homogenized using an ultrasonic cell disintegrator for 40 min, the liposome was successively filtered using 0.45 µm and 0.22 µm millipore membrane for sterilization, and stored at 4°C for further experiment.

CS‐An‐Lip is prepared by electrostatically depositing a cationic chitosan layer onto the surface of an anionic liposome loaded with anthocyanins, in short, by dissolving chitosan at pH 5.5. A chitosan solution was prepared in a 0.1% (v/v) acetic acid solution, and 0.45 μm was filtered to remove insoluble impurities. The An‐Lip dispersion was slowly added to an equal volume of chitosan solution and further mixed for 30 min with magnetic stirring at 25℃. Leave the resulting mixture still overnight at 4°C. The excess acetic acid of the chitosan‐coated liposomes was removed by centrifugation at 3,000 rpm for 3 min; then, the precipitated liposome pellet was resuspended in a 5% dextrose solution.

### 2.3 Experimental design

RSM was developed to acquire the optimal preparation conditions by describing the relationships between the variables and the response (Luo et al., 2014). Three levels (determined in preliminary experiments) were studied for each of the three independent variables: ratio of SP to Cholesterol (A, w/w), ratio of SP to BA (B, w/w), and rotary evaporation temperature (C) (Table 1). Details of the 17 design points are shown in Table 2. All the experiments were developed in triplicate. The experimental data were subjected to multiple regression analysis to obtain the adjusted polynomial equation. ANOVA was done to identify the significant coefficients. The three‐dimensional and contour graphs were built to identify the optimal combination of independent variables. For the analysis of the results, the Design‐Expert V 8.6.0 software was used.

**TABLE 1:** Levels and code of variables chosen for Box–Behnken design

Level	Factors		
	A	B	C
−1	3:1	10:1	30°C
0	4:1	20:1	40°C
1	5:1	30:1	50°C

**TABLE 2:** Arrangement and results of central composite design

No.	A	B	C	EE%
1	4	20	40	82.12
2	4	30	30	78.73
3	3	30	40	78.74
4	4	20	40	82.08
5	4	20	40	81.96
6	4	30	50	80.00
7	5	10	40	75.98
8	5	30	40	77.87
9	5	20	50	77.87
10	4	10	30	76.82
11	4	10	50	78.42
12	3	10	40	77.63
13	4	20	40	82.13
14	3	20	30	77.31
15	5	20	30	76.83
16	3	20	50	78.22
17	4	20	40	81.83

### 2.4 Characterization of An‐Lip

#### 2.4.1 Encapsulation efficiency (EE%) and loading capacity (LC%) of An‐Lip and CS‐An‐Lip

The encapsulation efficiency (EE) is defined as the ratio of An‐Lip and CS‐An‐Lip entrapped in liposomes to that in the delivery system, which is an important parameter for nanoliposomes when defined as delivery systems (Peng et al., 2010). It was calculated to determine the concentration of entrapped An‐Lip and CS‐An‐Lip in nanoliposomes and unentrapped An‐Lip and CS‐An‐Lip in the aqueous phase. The An‐Lip and CS‐An‐Lip were separated from the aqueous phase using a freezing centrifuge (5810R, Eppendorf). A 0.5 mL nanoliposome suspension was taken and spun at 10,000 rpm for 30 min at 4°C. The same suspension was ruptured using a certain volume of ethanol, and the total amount of An was determined spectrophotometrically. The percentage of encapsulation efficiency (EE) was calculated according to Equation (Zhao et al., 2016):

(1) $$\text{Encapsulation efficiency}\;\left(\text{EE}\%\right)=W_{\text{en}}/W_{\text{total}}\times 100\%$$

where W _en_ is the amount of free An, and W _total_ is the total amount of An present in 0.5 mL of nanoliposomes (W _total_ and W _en_ were measured by spectrophotometer and then calculated).

#### 2.4.2 Determination of particle size, polydispersity index, and ζ‐potential

The particle size, polydispersity index, and ζ‐potential of An‐Lip were determined using a NanoBrook 90Plus Zeta at 25°C (Tang et al., 2000). About 200 µL of each sample was taken and dispersed in deionized water to a final volume of 3 mL for the determination process. All data were calculated as the average of at least triplicate measurements.

#### 2.4.3 Surface morphology observation

An‐Lip prepared at the optimum conditions was studied using a field‐emission scanning electron microscope (SEM, Model H‐7650, Hitachi, High‐Technologies Co, Ltd.) operating at an accelerating voltage of 5 kV using a secondary electron detector 14. Take a small amount of sample powder deposited onto silicon wafer mounted on aluminum specimen stubs, and sputter coating (5 nm) with platinum to observe.

#### 2.4.4 Fourier transform infrared (FTIR) analysis

Fourier transform infrared (Perkin Elmer) was used to confirm the binding of anthocyanins to liposomes. The samples were mixed with 10% mannitol (used as drying protectant) to protect the structural integrity of liposomes (Guldiken et al., 2017), Then, the mixture was freeze‐dried and lay the sample flat in the sample tank for analysis. The scanning range was set at 4,000 cm^−1^ to 400 cm^−1^. Each spectrum was obtained by 64 scans, and the resolution was set at 4 cm^−1^. (Katouzian and Taheri, 2021).

### 2.5 Stability of the An‐Lip and CS‐An‐Lip during the storage

#### 2.5.1 Storage environment stability

The stabilization effect of An‐Lip and CS‐An‐Lip was assessed by measuring the change of A_520_ and release rate of anthocyanin with time at room temperature and 4°C.

#### 2.5.2 Color changes of anthocyanin liposome

The tristimulus color coordinates of the anthocyanin liposomes were measured using an instrument colorimeter (Chroma Meter CR‐400, Konica Minolta). The instrument is calibrated with a standard black and white plate. The measured parameters are L*(white/black), a* (red/green), and b* (yellow/blue).

### 2.6 In vitro release of anthocyanins in liposomes

The in vitro release kinetics of anthocyanins from liposomes was performed by the previously described dialysis bag method (Li et al., 2017) with minor modifications. Briefly, 2 mL liposome samples were transferred to a dialysis bag (*Mw* cutoff 8–14 kDa, Solarbio). The dialysis bag was then incubated in 50 mL phosphate buffer (pH 7, 5 mM) release medium at 10,956 *g* in a shaker for 72 h at 37°C. At specific time intervals, remove 2 mL of the release medium and replace with fresh medium. The release medium was diluted with MeOH to calculate the total cumulative amount of anthocyanins released from the liposomes. Anthocyanin content was measured at various sampling times at 520 nm using a spectrophotometer.

### 2.7 Simulation of in vitro digestion for infusions

A simulated gastrointestinal (GIT) model was used to assess the bioavailability of anthocyanins in liposomes. As described elsewhere (Li et al., 2018) and with some modifications, a simplified model of simulated GIT was established, including the stomach and small intestine. The entire digestion process was carried out in a 37°C water bath shaker (DHSZ, Jiangsu Taicang Experimental Equipment Co.). All solutions were preheated at 37°C prior to mixing.

#### 2.7.1 Stomach phase

The simulated gastric fluid (SGF) was prepared by dissolving NaCl (2 g), concentrated HCl (7 mL), and pepsin (3.2 g) into distilled water (1 L). The liposomes were mixed with SGF in a volume ratio of 1:1. The pH of the simulated gastric phase was adjusted to about 1.5, and this digestion step took 4 h.

#### 2.7.2 Small intestine phase

The simulated intestinal fluid (SIF) was prepared by dissolving K_2_HPO_4_ (6.8 g), NaCl (8.775 g), and pancreatin (3.2 g) into distilled water (1 L). Before the addition of SIF, the pH of mixtures taken from simulated stomach phase must be adjusted to 6.8 approximately, because the extremely acid environment could inactivate the pancreatin. Mixtures taken from simulated stomach phase were also mixed with SIF at a volume ratio of 1:1. The pH was adjusted to 7.0, and this digestion period took 4 h.

### 2.8 Antioxidant activity

The DPPH and ABTS assays of anthocyanins liposomes were measured in accordance with a previously described method with slight modifications (Samsonowicz et al., 2019; Xu et al., 2019). 0.1 mmol L^−1^ DPPH stock solution was prepared by dissolving 0.1984 g of DPPH with anhydrous methanol to 50 mL. The samples were prepared into different concentrations of sample solutions with anhydrous methanol. 1 mL of sample solutions with different concentrations was accurately absorbed and added into 3 mL of 0.1 mmol L^−1^ DPPH stock solution respectively, and kept away from light for 30 min at room temperature. Using deionized water as the reference solution and VC as the control group, the absorbance was measured at 517 nm, and the DPPH radical scavenging rate was calculated according to formula.

EtOH was used as the control. DPPH radical scavenging activity was calculated by using the following equation:

(2) $$\text{DPPH radical scavenging activity}=\left(A_{517}\text{Blank}‐A_{517}\text{Sample}/A_{517}\text{Blank}\right)\times 100\%$$

where A_517_ is the absorbance measured at 517 nm.

Accurately, suck 1 mL of sample solutions of different concentrations, successively add 1 mL of 9 mmol L^−1^ FeSO_4_ solution, 1 mL of 9 mmol L^−1^ salicylic acid solution and 1 mL of 8.8 mmol L^−1^ H_2_O_2_ solution, shake well, react in a 37°C water bath for 30 min, take deionized water as the reference solution and VC as the control, set 3 parallel in each group, and measure the absorbance value at 734 nm. ABTS radical scavenging activity was calculated by using the following equation:

(3) $$\text{ABTS radical scavenging activity}=A_{734}\text{Blank}‐A_{734}{\text{SampleA}}_{734}\text{Blank}\times 100\%$$

where A_734_ indicates the absorbance measured at 734 nm.

### 2.9 Statistical analysis

Data were analyzed by Design‐Expert V 8.6.0 software where appropriated. Results were described as means ± SE. One‐way analysis of variance (ANOVA) was used to determine statistically significant difference among groups, and means of every two different groups were detected with test.

## 3 RESULTS AND DISCUSSION

### 3.1 Experimental design and data analysis

The combined effects of three factors with three levels on EE% were listed in Table 2 and were evaluated by a 17‐run BBD for parameter optimization.

The fitted equation for predicting the maximum EE% was given as follows: Y = +82.02−29A+94B+0.60C+0.46AB+0.033AC−0.083BC−2.57A_2_−1.64B_2_−1.90C_2_.

**FIGURE 1:**
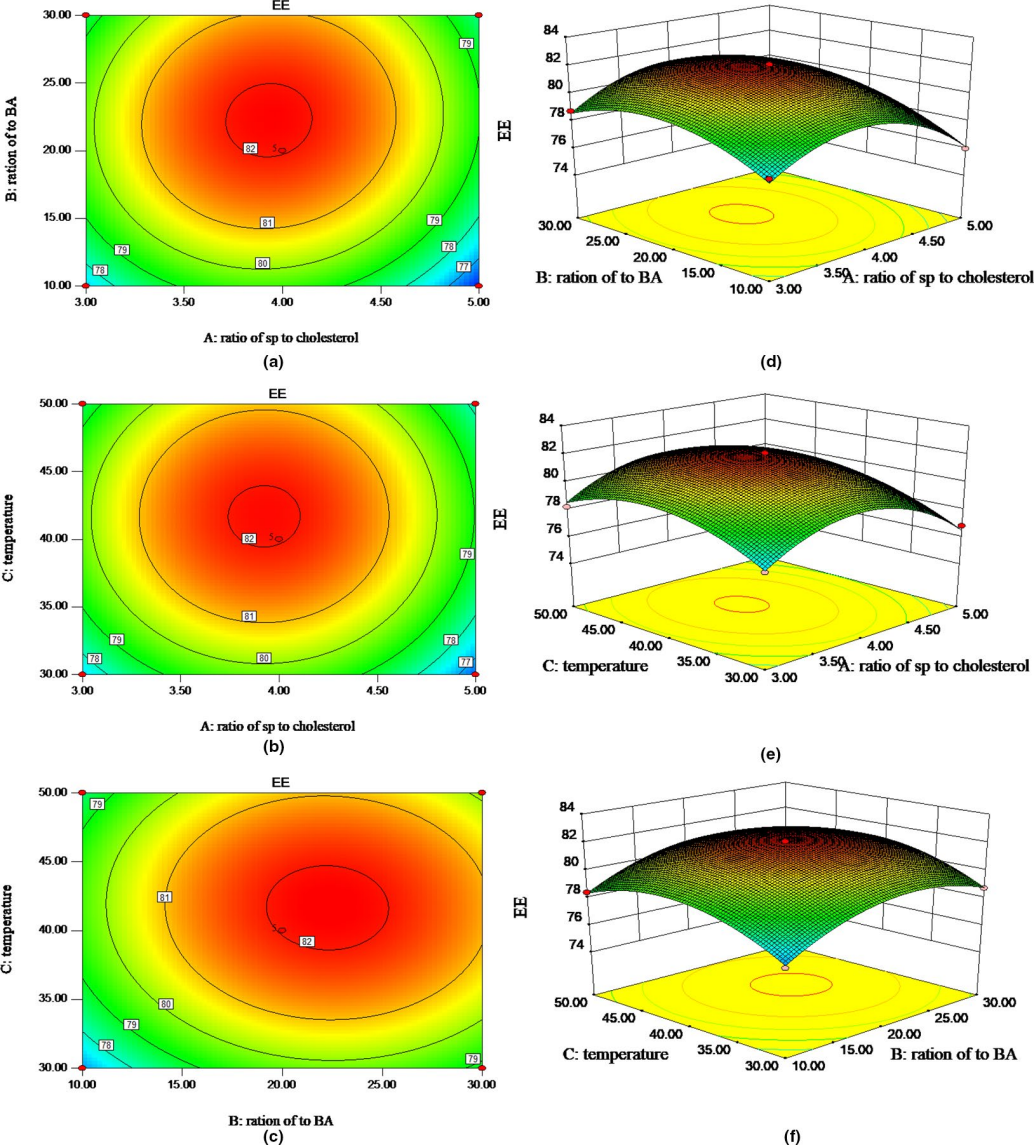
3‐D response surface plots and 2‐D contour plots showing effects of various parameters on EE%. Effects of SP to cholesterol ratio (a,d), rotary evaporation temperature (b,e), and SP to BA ratio (c,f) on the encapsulation efficiency of anthocyanin liposomes

To show the type of interactions between the independent variables and the relationship between responses and levels of each variable, the response surface plots for EE% are presented in Figure 1.

#### 3.1.1 Effect of the independent variables on the encapsulation efficiency

In this study, A, B, C, AB, A_2_, B_2_, and C_2_ are significant model terms. The Model *F*‐value of 213.58 implies the model is significant, which confirmed that the model could correctly explain and predict the results in the experiments. The *R* ^2^ value and *R* ^2^ad could indicate that the regression models were fit for explaining the variability of response. These results show that the effects of above various factors on An‐Lip preparation are not simple linear relationship. The adjusted determination coefficient (*R* ^2^adj) is 0.9917, indicating that the model is suitable to represent the actual relationship between response and variables, that is, the model is in line with this study (Table 3).

**TABLE 3:** Analysis of variance

Sum of source	Squares	*df*	Mean square	*F* value	*p*‐value	significant
Model	71.82	9	7.98	213.58	<.0001	***
A‐SP: cholesterol	0.66	1	0.66	17.70	.0040	**
B‐SP: BAL	7.11	1	7.11	190.22	<.0001	***
C‐ temperature	2.90	1	2.90	77.73	<.0001	***
AB	0.84	1	0.84	22.41	.0021	**
AC	4.22	1	4.22	0.11	.7456	
BC	0.027	1	0.027	0.73	.4215	
A^2^	27.83	1	27.83	744.82	<.0001	**
B^2^	11.27	1	11.27	301.55	<.0001	**
C^2^	15.13	1	15.13	405.03	<.0001	**
Residual	0.26	7	0.037			
Lack of fit	0.20	3	0.065	4.00	.1067	Not significant
Pure error	0.065	4	0.016			
Cor total	72.08	16				
*R* ^2^ = 0.9964, Adj *R* ^2^ = 0.9917, Pred *R* ^2^ = 0.9550						

* *p* < .05 (significant)

** *p* < .01 (highly significant)

*** *p* < .001 (extremely significant).

The corresponding response surface figure and contour map were drawn with the binomial fitting model. The results showed that with the increase of SP, the ratio of SP to cholesterol, and the ratio of SP to BA, EE% first increased and then decreased. This phenomenon may be related to the saturation degree of liposomes. Cholesterol acts as a "flow buffer" in liposome system. Higher than the phase transition temperature, the lipid bilayer can be arranged closely, thus reducing the drug leakage and increasing the EE%, the asymmetry, permeability, and rigidity of lipid bilayer membrane increased with the increase of cholesterol dosage, which resulted in drug penetration and decreased EE% (Li et al., 2018). In addition, the steeper the surface was, the more significant the effect indicated, so ratio of SP to cholesterol and ratio of SP to BA were the two main effects on encapsulation efficiency in this experiment. Surface openings in three‐dimensional plots were downward. Encapsulation efficiency was on the rise as the growth of each factor, and then gradually reduced when it reached the peak. The peak value was also the maximum encapsulation efficiency point in these three factors (Figure 1).

The regression equation is analyzed to obtain the best technological conditions and the predicted value of encapsulation efficiency. The optimum technology is the ratio of SP to cholesterol is 3.97:1, the ratio of SP to BA is 22.81:1, and rotary evaporation temperature is 41.53°C. The predicted value was 82.2062% under this condition. Considering the actual production situation, adjusted ratio of SP to cholesterol was 4:1, ratio of SP to BA was 20:1, and rotary evaporation temperature was 40℃ as the optimum technology.

#### 3.1.2 Validation of the optimized model

Box–Behnken design has the characteristics of good predictability, intuitive results, and simple operation. It can optimize the process parameters faster and more efficiently with the least number of experiments. At present, it has been widely used in drug prescription screening. In this experiment, Box–Behnken combined with response surface method was used to optimize the formulation of lipid preparation, which greatly reduced the workload, successfully constructed the model of the mass ratio of phospholipid to cholesterol, the volume ratio of phospholipid to anthocyanin and the effect of temperature on the entrapment efficiency, intuitively predicted the optimal formula, prepared three batches of samples according to the optimized conditions, measured their entrapment efficiency respectively, and carried out validation experiments, The predicted value of the optimal formulation liposome entrapment efficiency predicted by the model is 82.21%, the average value of the measured entrapment efficiency is 82.13%, and the deviation is 0.08%, indicating that the prediction of this model is accurate and reliable, the reproducibility of the formulation process is good, the particle size of the prepared liposome meets the requirements, the entrapment efficiency is stable, and it is suitable for the preparation of An‐Lip.

### 3.2 Characterization of An‐Lip

#### 3.2.1 DLS, Zeta, PDI, and TEM characterization

**FIGURE 2:**
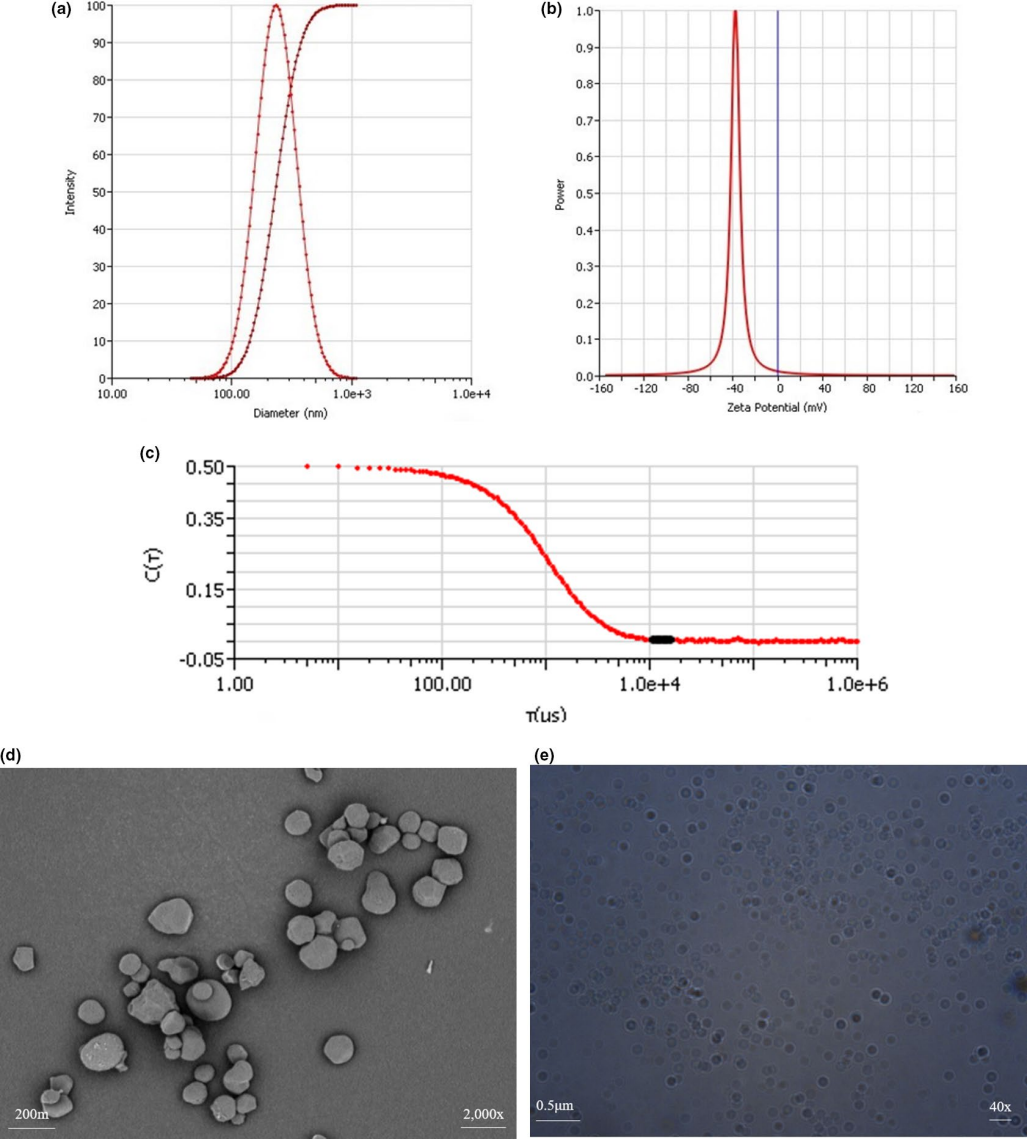
TEM image and particle size distribution of anthocyanin liposomes. The results showed the following: (a) the particle size distribution of anthocyanin liposomes (according to intensity); (b) the potential distribution of anthocyanin liposomes; (c) the relationship between the scattered light intensity and time of anthocyanin liposomes; (d) the TEM image of anthocyanin liposomes (scale = 200 nm); (f) the electron microscope image of anthocyanin liposomes (scale = 0.5 μm)

An‐Lip presents a standardized particle size distribution curve, showing the variation between particle sizes. The main particle sizes of An‐Lip range from 100 nm to 300 nm (Figure 2a). Zeta potential was used as an indicator of the physical stability of liposomes. The zeta of An‐Lip prepared in this study was −31.05 ± 1.0 mV (Figure 2b), indicating that the liposomes were negatively charged and the system was stable. Figure 2c shows the relationship between the intensity of scattered light and time in An‐Lip, as well as the relationship between time = t and the intensity of scattered light signal at different time points. The following points can be drawn from the correlation graph: first, the relationship between light intensity and time decreases with the passage of time, and finally tends to zero due to the random Brownian motion of liposome particles. Second, the final smooth baseline indicates no precipitation in the sample. Finally, the correlation diagram also shows that the particle size is relatively large, because the signal changes slowly, and the correlation lasts for a long time (stable period) before attenuation. TEM and optical microscope images showed that An‐Lip was spherical with a size of about 200 nm and formed vesicle structure (Figure 2d–f). The spherical shape of An‐Lip was similar to that of liposomes obtained in previous studies (Tang and Pikal, 2004; Guldiken et al., 2018). The PDI values of nanoliposomes were 0.112–0.255. The PDI value of An‐Lip was 0.237. In general, PDI measures particle size distribution from homogeneous to heterogeneous (0–1.0), where homogeneous particles are considered to be in the range of 0 to 0.3 (Zhao and Temelli, 2017). Therefore, the nanoliposome carrier developed in this study has uniform dispersion.

#### 3.2.2 Characterization of An‐Lip and CS‐An‐Lip

As shown in Table 4, the coating of chitosan significantly increased the particle size and polydispersity index of the liposome. A higher chitosan content results in a larger particle size increase, which also indicates the formation of an additional chitosan layer. In the anthocyanin liposomes, the particle size increase of chitosan coating was more pronounced than that of empty liposomes. This indicates that the thicker layer is composed of negatively charged anthocyanins and positively charged shells. Although chitosan forms an interaction between the layers, the thicker layer does not seem to indicate that more anthocyanins are incorporated into the chitin liposomes because the chitin coating does not increase the load of anthocyanins in the liposomes.

**TABLE 4:** Characterization of anthocyanin liposomes coated with different chitosan concentrations

Liposomes	EE (%)	Zeta potential (mV)	Mean particle size (nm)	Polydispersity (PDI)
Blank liposome	–	−25.16 ± 1.31	156.153 ± 2.230	0.217 ± 0.033
An‐Liposomes	74.730 ± 1.255	−29.19 ± 2.09	191.373 ± 3.636	0.237 ± 0.042
Chitosan0.05%	79.783 ± 2.292	26.11 ± 0.93	242.650 ± 10.373	0.202 ± 0.056
Chitosan0.1%	84.403 ± 0.829	31.91 ± 0.48	233.916 ± 13.090	0.220 ± 0.027
Chitosan0.2%	81.510 ± 1.099	26.94 ± 1.24	280.940 ± 12.782	0.244 ± 0.024
Chitosan0.3%	77.740 ± 0.653	29.30 ± 0.76	312.416 ± 7.148	0.260 ± 0.019

#### 3.2.3 FTIR analysis

**FIGURE 3:**
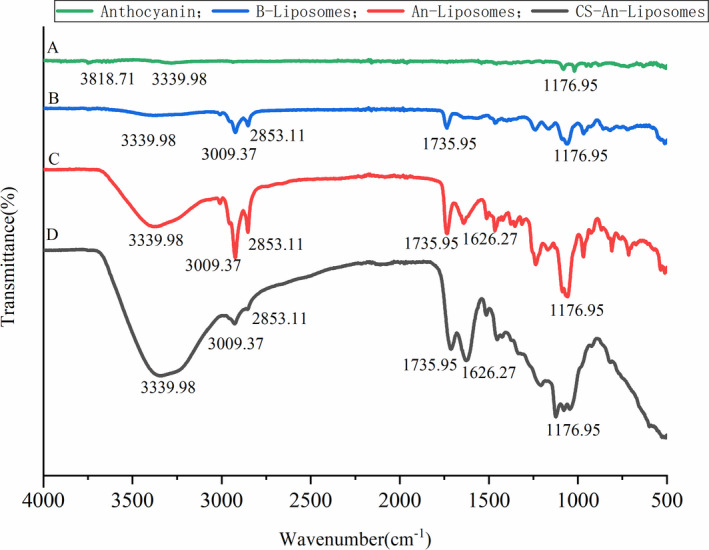
IR spectra of anthocyanin (a), blank liposome (b), An‐liposome (c), and CS‐Anthocyanin‐liposome (d)

**FIGURE 4:**
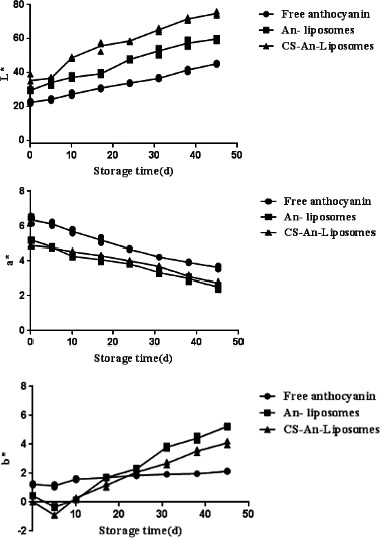
Effect of storage time on the color of free anthocyanins, anthocyanin liposomes, and chitosan‐coated anthocyanin liposomes during storage at 4. (a) L * (light); (b) a * (red‐green); and (c) b * (yellow‐blue)

In order to further confirm the successful embedding of anthocyanins, Fourier transform infrared spectroscopy (FTIR) was used to characterize. In the range of 4,000–2,500 cm^−1^, as shown in Figure 3a, An has an absorption peak at 3818–3339 cm^−1^, and the stretching vibration of hydroxyl group occurs, but the absorption peak is obviously weakened, which is due to the reduction of carbonyl group in blueberry anthocyanins structure after purification (Cai et al., 2019). The skeleton vibration of benzene ring was mainly concentrated around 1596.60 cm^−1^ with only one absorption peak, and the unsaturated C‐H deformation vibration absorption peak appeared at 1176.96 cm^−1^ of fingerprint region. Figure 4b shows blank liposomes, and the absorption peaks at 2853.11 cm^−1^ and 3009.37 cm^−1^ are caused by the C‐H Tensile vibration of liposome shell, while the absorption peaks at 1735.95 cm^−1^ and 1176.95 cm^−1^ correspond to C = O and C‐O‐C Tensile vibration. In Figure 4c, for An‐Lip, the characteristic peaks were observed at around 3339.98 cm^−1^ (O‐H stretching), 3009.37 cm^−1^ (C = O stretching vibration), and 1626.27 cm^−1^ (C‐H stretching vibration). The peaks observed at 1300–1100 cm^−1^ refer to the PO2 symmetric and asymmetric stretching vibration of a phospholipid, respectively, The An‐Lip showed almost similar spectra to An which could be due to the well‐encapsulation of An into the aqueous core of Lip. It is worth noting that the FTIR spectrum of An‐Lip is similar to that of blank Lip, and there is no characteristic absorption peak of An, which indicates that the coating mode of liposomes is physical coating. In Figure 4d, the characteristic spectrum of CH shows that the typical polysaccharide has a wide and strong peak at 3500–3000 cm^−1^. The typical peak appears at 3339.98^−1^ cm (OH stretching vibration of CH) and 1735 cm^−1^ (amino stretching vibration of CH). After the formation of CH layer, the stretching vibration of C = O and C‐H shifts. The change of specific atomic and / or ionic signal state may be the result of hydrogen bond enhancement or new hydrogen bond formation, which is obviously related to biopolymer deposition (Sarabandi et al., 2019). The possible mechanism is that the positively charged amino group of CH interacts with liposomes, resulting in the successful deposition of CH on CH‐An‐Lip (Katouzian and Jafari, 2016). The nanoliposomes were coated by hydrophobic interaction or hydrogen bonding. Therefore, FTIR analysis results can further confirm the successful coating of An.

### 3.3 Stability of the chitosan coating of anthocyanin liposomes

#### 3.3.1 Storage environment stability

The two liposomes were accurately transferred and placed at room temperature for sampling at 0, 1, 2, and 3 months. The encapsulation efficiency, drug‐loading rate, zeta potential, and particle size were used as evaluation indexes to investigate the shell aggregation. The results are shown in Table 5. As is clear from the Table 5, the liposome has a lower degree of change in the encapsulation efficiency and the drug‐loading rate at 4°C; thereby, it can be seen that the prepared liposome is suitable for storage under 4°C environments. In addition, under the same environment, the encapsulation efficiency of chitosan‐modified anthocyanin liposomes was higher than that of unmodified liposomes, and the drug‐loading rate decreased slowly. Due to the incorporation of positively charged chitosan, the potential of anthocyanin liposomes is negatively converted to a positive charge, which also increases the charge of the liposome.

**TABLE 5:** Physical stability of anthocyanin liposomes and chitosan‐coated liposomes at 4°C and 25°C

T (℃)	Time (month)	An‐Liposomes				CS‐An‐Liposomes			
		EE%	LC%	Particle (nm)	ζ‐potential (mV)	EE%	LC%	Particle (nm)	ζ‐potential (mV)
4	0	82.38 ± 3.18	4.23	191.373 ± 3.636	−27.83 ± 3.12	84.38 ± 3.18	4.73	204.28 ± 2.932	34.29 ± 2.32
	1	78.27 ± 1.16	3.35	203.256 ± 1.287	−25.93 ± 7.18	80.18 ± 0.182	4.11	217.73 ± 0.182	32.12 ± 0.21
	2	75.28 ± 1.92	2.74	224.834 ± 0.280	−28.38 ± 1.93	79.91 ± 2.917	3.72	228.92 ± 4.283	35.23 ± 4.29
	3	73.95 ± 0.38	2.03	242.550 ± 2.178	−24.92 ± 3.82	76.56 ± 7.283	3.03	232.39 ± 2.932	29.37 ± 8.28
25	0	83.38 ± 3.18	4.23	191.373 ± 3.636	−27.83 ± 3.12	84.38 ± 3.18	4.73	204.28 ± 2.932	34.29 ± 2.32
	1	74.29 ± 5.93	3.02	235.324 ± 2.628	−26.97 ± 5.37	77.27 ± 2.921	3.89	238.92 ± 4.283	30.82 ± 4.25
	2	69.24 ± 0.23	2.14	251.28 ± 7.271	−17.26 ± 0.16	73.92 ± 7.432	3.08	258.39 ± 2.93	27.92 ± 6.83
	3	65.63 ± 2.99	1.67	301.554 ± 2.128	−13.72 ± 4.83	67.21 ± 4.82	2.57	273.28 ± 5.82	25.62 ± 3.28

#### 3.3.2 Color changes of anthocyanin liposome

The color changes shown by anthocyanins liposomes during storage at 20°C for 2 weeks were examined. The results are presented in Figure 4. The L*, a*, and b* values represent the measure of brightness, red or green and yellow or blue respectively (Jeon et al., 2015).

During storage, the L* values of An‐Lip and CS‐An‐Lip increased due to the incorporation of lipid material and chitosan (Figure 4a), but the loading of chitosan makes the L* value of anthocyanins slower than that of chitosan‐free liposomes. The a* values of free anthocyanins, An‐Lip, and CS‐An‐Lip decreased (Figure 4b), and anthocyanin liposomes increased with time. The change of An‐Lip in a* value is higher than that of CS‐An‐Lip, and it indicates that the color after chitosan modification during storage is more stable than the color of free anthocyanins and unmodified lipids. During storage, the b* values of the two forms of liposomes changed slightly, while the b* values of free anthocyanins were negligible (Figure 4c). After storage for 10 days, the b* value of the chitosan‐coated lipids is lower than that of the anthocyanin liposome without modification with chitosan. The results indicate that chitosan‐modified liposome encapsulation can delay the degradation of anthocyanins, but does not prevent the color stability of anthocyanins during storage.

### 3.4 In vitro release study

**FIGURE 5:**
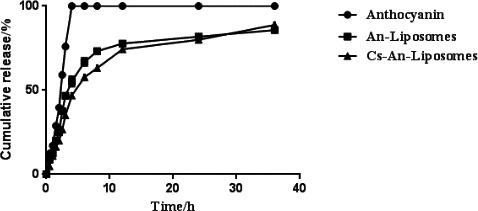
In vitro release curves of anthocyanins, An‐Lip, and CS‐An‐Lip at 4°C during 36 h

In order to effectively target organs and tissues passively, liposomes loaded with nutraceuticals should retain nutrients for a long time during the cycle. Therefore, the anthocyanin release behavior of the liposomes was evaluated using a dialysis bag diffusion technique. The in vitro release profile of anthocyanins from An‐Lip and CS‐An‐Lip is shown in Figure 5. CS‐An‐Lip has a slower rate of anthocyanins release than An‐Lip. For example, after 72 h, only about 59% of the anthocyanins were released from CS‐An‐Lip, while about 83% of the anthocyanins were released from An‐Lip. These results indicate that the presence of chitosan can inhibit the release of anthocyanins from liposomes, which may be beneficial for extended release applications. It is speculated that chitosan increases the affinity of anthocyanins for the hydrophobic domains within the liposomes, thereby reducing their release tendency (Christodouleas et al., 2014).

### 3.5 Simulation of in vitro digestion for infusions

**FIGURE 6:**
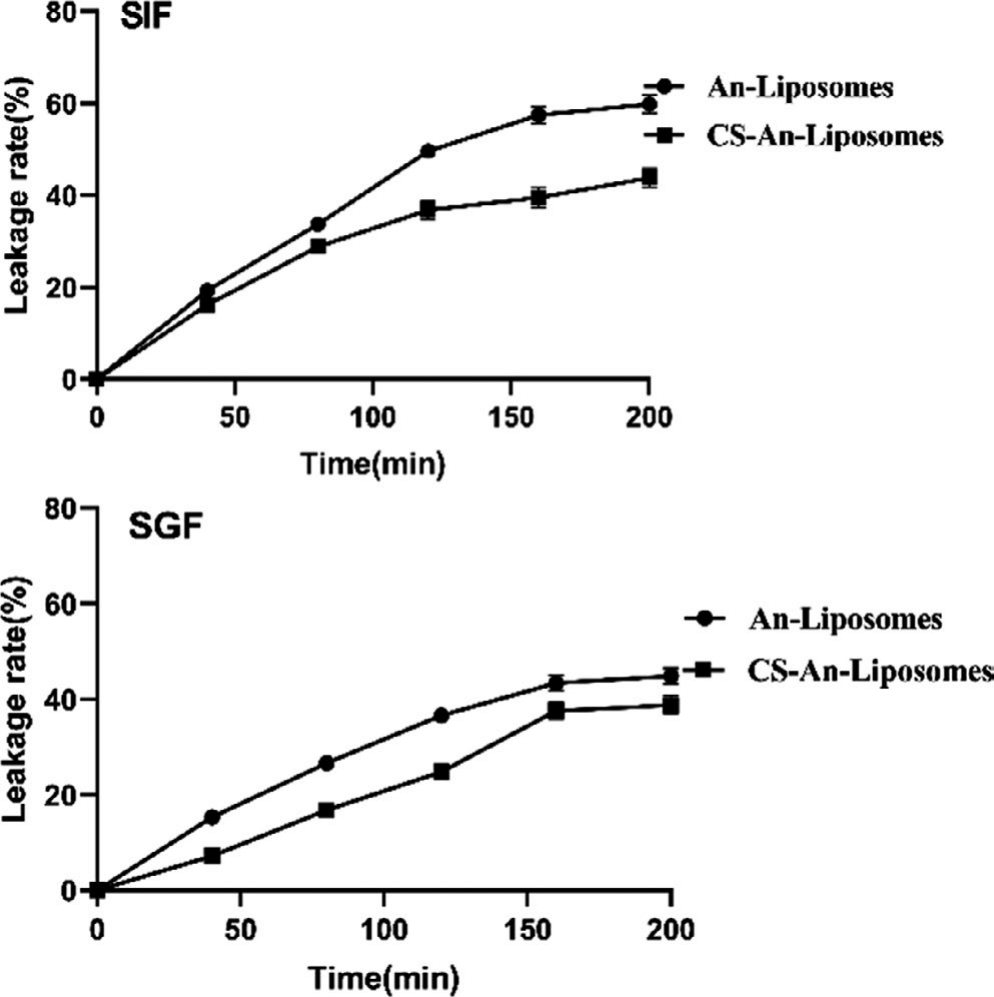
Digestion of free anthocyanins, An‐Lip, and CS‐An‐Lip in stomach phase(A) and small intestine phase(B)

It can be seen from Figure 6 that the leakage rate of the unmodified liposome tends to be stable at 120 min, while the chitosan‐modified liposome begins to stabilize at 90 min and is digested in an in vitro simulated intestinal environment for 2.5 h. After 2.5 h of digestion, the unmodified liposome anthocyanin leakage rate was 54.85%, while the chitosan‐modified liposome had an anthocyanin leakage rate of 43.76%. It was shown that the stability of chitosan‐modified liposomes and unmodified liposomes in the in vitro simulated intestinal environment was significant. The hydrolysis of phospholipids by pancreatic enzymes in the intestinal juice and the solubilization of bile salts are the main reasons for the rapid and massive release of anthocyanins. After the liposomes are coated with chitosan, the rapid release of anthocyanins is inhibited, and the release is more likely to occur slowly and continuously. Studies have shown that the coating formed by chitosan on the surface of liposomes can effectively inhibit the penetration of lipase and bile salts into the lipid bilayer and enhance the stability of liposomes in the intestinal fluid environment (Zhao et al., 2015).

It can be seen from Figure 6 that the leakage rate of anthocyanins is time‐dependent. The anthocyanin leakage rate increases with time, and the leakage rate tends to be stable at 120 min. It was also found that the stability of the liposome sample in the stomach environment was significantly higher than in the intestinal environment. This indicates that since the pH of SGF is 2, anthocyanins are very stable under acidic conditions. After 2.5 h of digestion, the unmodified liposome anthocyanin leakage rate was 45.85%, and the chitosan‐modified liposome leakage rate was 35.76%, which significantly improved its stability in the stomach. This indicates that the polysaccharide‐modified liposome can effectively inhibit the release of anthocyanins in gastric juice and help to reduce its destruction by enzymes and acids in the stomach.

### 3.6 Antioxidant activity

**FIGURE 7:**
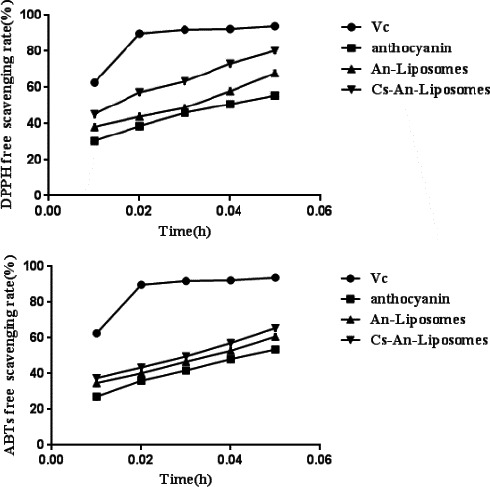
Activity of DPPH(A) and ABTS(B) free radicals to remove free anthocyanins, An‐Lip, CS‐An‐Lip, and Vc

Several chemical analysis methods have been established to evaluate the oxidation/reduction potential of plant compounds (Liang et al., 2017; Narod and Nazarali, 2014). DPPH and ABTS analyses are authoritative methods for measuring antioxidant activity and are used in this study to compare the antioxidant activity of free anthocyanins, An‐Lip, CS‐An‐Lip, and Vc (Figure 7).

The DPPH assay of the sample showed a dose‐dependent manner (Figure 7a). The Vc group was the total control group, and the clearance rate of all samples increased with the increase of its concentration. The clearance rate of anthocyanin liposomes and chitosan‐modified anthocyanin liposomes is higher than that of free anthocyanins. The clearance rate of the sample follows the order of Vc > CS‐An‐Lip > An‐Lip > free anthocyanin. These results indicate that liposomes can increase the DPPH clearance rate of anthocyanins compared with free anthocyanins, and chitosan‐modified liposomes are more effective. Our previous study reported similar results (Moretton et al., 2012).

ABTS analysis showed that the clearance rates of samples at different concentrations (0.01, 0.02, 0.03, 0.04, and 0.05 mg/mL) were dose dependent (Figure 7b). With Vc as the control group, the clearance rate of all samples increased with the increase of its concentration. The clearance rate of An‐Lip is higher than that of free anthocyanins. These results indicate that liposomes can increase the ABTS free radical scavenging rate of anthocyanins.

## 4 CONCLUSIONS

In this study, the formulation of anthocyanin liposomes was optimized by response surface methodology, and nanoliposomes with high entrapment efficiency were successfully prepared. On this basis, chitosan was added to improve its stability and functionality. It was characterized by a variety of analytical equipment. Through the experimental design, the best sample is selected based on the central composite design. The average particle size of the best nano‐liposome was less than 300 nm, and the zeta potential showed that the system was stable. FTIR spectra showed that there were electrostatic interactions and hydrogen bonds between phospholipid polar groups, chitosan amine part, and main anthocyanin extract polyphenols. In addition, the results of environmental stability analysis showed that the addition of chitosan could stabilize the color characteristics and content of anthocyanins under certain conditions. In vitro release and simulated gastrointestinal digestion experiments showed that the addition of chitosan not only prolonged the release of anthocyanins, but also prolonged the retention time of anthocyanins in vivo. In addition, the antioxidant activity test showed that CS‐An‐Lip could improve the antioxidant activity of anthocyanins. Therefore, the combination of biopolymer and chitosan on the Lip surface is a promising method, which has great application potential in food, dietary supplement, and pharmaceutical industry. In further research, it is recommended to implement the developed loaded chitosan to strengthen/enrich food and test the acceptability of the product through sensory evaluation. The interesting results obtained in this study will pave the way for the application of these nanostructures in different food systems.

## CONFLICT OF INTEREST

The authors declare that they do not have any conflict of interest.

## ETHICAL APPROVAL

This study does not involve any human or animal testing.

## INFORMED CONSENT

Written informed consent was obtained from all study participants.

## Data Availability

The data that support the finding of this study are available from the corresponding author upon resonable request.
